# Access to primary care in adults in a provincial correctional facility in Ontario

**DOI:** 10.1186/s13104-016-1935-4

**Published:** 2016-02-29

**Authors:** Samantha Green, Jessica Foran, Fiona G. Kouyoumdjian

**Affiliations:** Department of Family and Community Medicine, St. Michael’s Hospital, 30 Bond Street, Toronto, ON M5B 1W8 Canada; Department of Political Science, McMaster University, Hamilton, ON Canada; Centre for Research on Inner City Health, St. Michael’s Hospital, Toronto, ON Canada

**Keywords:** Detention, Prison, Incarceration, Primary care

## Abstract

**Background:**

Little is known about access to primary care either prior to or following incarceration in Canada. International data demonstrate that the health of people in prisons and jails is poor, and access to primary care in the community may be inadequate for incarcerated persons. We aimed to describe the primary care experience of adults in custody in a provincial correctional facility in Ontario in the 12 months prior to admission.

**Methods:**

We conducted a written survey, and invited all persons in the institution to participate, excluding those in segregation.

**Results:**

One hundred and twenty-five persons participated, 16.8 % of whom were women. The median age was 33. In the 12 months prior to admission to custody, 32.2 % (95 % CI 23.5–40.8 %) of respondents did not have a family doctor or other primary care provider and 48.2 % (95 % CI 38.8–57.6 %) had unmet health needs. Participants reported a mean of 2.1 (SD = 2.8) emergency department visits in the 12 months prior to admission.

**Conclusions:**

Study participants report a lack of access to primary care, a high mean number of emergency department visits, and high unmet health care needs in the 12 months prior to incarceration. Time in custody may present an opportunity for connecting this population with primary care and improving health.

## Background

International data show that the health of people who experience incarceration is poor, with a disproportionate burden of mental illness, infectious diseases, chronic diseases, and premature mortality [1–7]. Evidence suggests that at the time of release from custody health care needs are particularly great, as illustrated by data on mortality [8, 9], hospitalization, and emergency department use [10–12]. Primary care could address health and social needs at the time of release; however, access to primary care may be lacking for this population, as has been noted in research form the United States and United Kingdom [13, 14].

In Canada, about 38,000 adults are in custody in correctional facilities on any given day [15]. About 63 % of these persons are held in provincial or territorial custody, which means they have not yet been sentenced or they have been sentenced to less than 2 years in custody [16–18], while the remainder (those sentenced to 2 years or more) are held in federal facilities.

Consistent with international data, Canadian data reveal that people who experience incarceration in Canada have poor health across a range of health status indicators [19], but there is a lack of Canadian data on several aspects of health status including access to primary care in custody and in the community. There is overrepresentation in this population of characteristics associated with limited access to health care, including high rates of poverty, unemployment, homelessness and substandard housing, low education, male, young age, Aboriginal ethnicity, and substance use [20–28]. Specific individual-level mechanisms that could compromise access to health care in the community include having multiple competing priorities, not being able to track or follow through with scheduled appointments, or lacking a government-issued health card, which is required to access most health care. Difficulty navigating the health care system and discrimination by practitioners may compound these challenges.

There are also structural factors that contribute to worse health and may limit access to health care in this population and in other marginalized populations, including the intensification of neoliberal polices [29–35]. Situating questions about health justice and access within contemporary policy agendas in Canada, including recent changes to the criminal code, one can appreciate that many of the same conditions that produce health inequities through barriers to access also produce crime, and increasingly impact poor, racialized, and marginalized communities within Canada’s expanding policing and prison system. This introduces additional nuance and depth to how we can understand questions of barriers and access.

Access to primary care may thus be worse for persons who experience detention and incarceration than it is for the general population in Canada, in which 15 % of persons do not have a family physician [36]. Further, existing health care services may not be structured to adequately deal with this population with multiple social challenges and medical comorbidities.

Access to primary care has been shown to be instrumental for achieving good health [37]. Having a family doctor allows for earlier treatment of conditions, more preventive care, and better management of chronic disease [38–45]. Having better continuity of care and an established physician-patient relationship also contributes to better outcomes and improved patient satisfaction [46, 47]. Continuity of care is associated with a decreased likelihood of hospitalization [48] and lower emergency department use [38, 49, 50].

In the context of a population with poor health status and putatively poor access to primary care, we aimed to describe the primary care experience of adults in custody in a provincial correctional facility in Ontario in the 12 months prior to admission.

## Methods

We obtained approval for the study from the Hamilton Integrated Research Ethics Board and the Ontario Ministry of Community Safety and Correctional Services. All participants provided written informed consent. We conducted a written survey of men and women in a provincial correctional facility in southwestern Ontario. The facility has a daily census of over 500 men and 40 women, and admits any persons from the region who are admitted to custody, whether they have been sentenced or not. Participants were eligible to complete the survey if they were 18 years of age or older, were not in segregation, and could read and write in English.

Initially the survey was distributed to inmates at the time of admission. Due to low rates of survey distribution or uptake, we modified study procedures with the input of facility staff and inmates. Two of the authors presented the survey to the group of inmates in each living area (“range”) in the facility, and distributed the survey to any interested persons in each range. We presented and distributed the survey a second time after 3 months on ranges with a high turnover rate. At the time of the survey distribution, staff explained that persons who had previously participated should not participate again.

Inmates were asked to review and complete the consent form and survey within the subsequent days. Surveys were returned in a sealed envelope to the study staff.

The survey included demographic questions as well as questions about health care access and health status, using validated questions from the Canadian Survey of Experiences with Primary Health Care [51] to allow comparability of results with data for the general population. See Appendix for a copy of the survey. We defined access to primary care as an affirmative response to the question: “Do you have a regular primary health care provider, such as a doctor or a nurse?” Self-rated health was a categorical variable, with options excellent, very good, good, fair, or poor. We also asked about whether participants had ever been diagnosed with chronic conditions that we hypothesize are prevalent in this population based on empirical data from other jurisdictions [3]. With no existing data on access to primary care in a Canadian prison or jail population, we used the rate of Canadians who do not have a family doctor, or 15 %, to calculate our sample size [36], with a margin of error of 7 % and a confidence level of 95 %. We used Stata 12 to analyze our data.

## Results

Five surveys were completed after distribution on admission, and 125 surveys were completed after distribution on the ranges; the response rate was 36 % with the revised survey distribution procedure (125/344). Twenty-one participants (16.8 %) indicated that their gender was female, while four participants (3.2 %) did not specify their gender. The median age was 33, the standard distribution (SD) was 10.2, and the range was 18–64 years. Almost half of participants (46.4 %, n = 58) had not completed high school.

Thirty-two point two percent (37/115, 95 % CI 23–40.8 %) of respondents reported that they did not have a family doctor or other primary care provider in the 12 months prior to admission to custody, including 5 of 20 women and 31 of 94 men. Those with no primary care provider indicated several reasons for not having a regular primary health care provider from a list of possible reasons, as shown in Table 1. Additional responses provided by participants were “always in and out of jail,” “[b]ecause I’m on methadone they won’t take me as a patient,” being fired from a practice because “I told [my doctor] off,” “No doctor will take me,” and “no ID.”

**Table 1:** Reported reasons why participants don’t have a regular primary health care provider, N = 37

Reason	Number (%)
I had a family physician who left or retired	9 (24.3)
There are no doctors available in the area	8 (21.6)
Doctors in the area are not taking new patients	8 (21.6)
I don’t know how to find one	7 (18.9)
I haven’t tried to contact one	2 (5.4)
I am in good health and I don’t need one	2 (5.4)

Participants could select more than one reason from a list of possible reasons, or specify another reason

Thirty-one participants (26.5 %) rated their health as excellent or very good, 44 (37.6 %) as good, and 42 (35.9 %) as fair or poor, of 117 question respondents.

Of 112 people who responded to the question, 48.2 % (95 % CI 38.8–57.6 %) reported unmet health needs in the 12 months before admission to custody. Table 2 shows the barriers in meeting their needs that participants identified from a list. Other barriers identified by participants were: not having a family physician and not knowing how to access care, no reminder call on a long wait, “Doctors don’t listen, I am judged by criminal record,” their lifestyle making it difficult to access care in terms of hours when awake, not being given time to address all problems during an appointment (even urgent ones), not being able to get a health card number, drug addiction and “head problems,” not having sought care because of feeling looked down upon because of addiction, and concerns about catching or flu or other infection at a health care facility.

**Table 2:** Reasons why participants didn’t get their health care needs met in the past year, N = 54

Reasons	Number (%)
The waiting time was too long	22 (40.7)
I had transportation problems	9 (16.7)
I had personal or family responsibilities	8 (14.8)
Care was not available when I needed it	8 (14.8)
I didn’t get around to it/didn’t bother	8 (14.8)
I didn’t know where to go	7 (13.0)
I dislike doctors/I felt afraid	5 (9.3)
I was too busy	4 (7.4)
I decided not to seek care	4 (7.4)
Cost	3 (5.6)
I felt the care would be inadequate	2 (3.7)
Care was not available in the area	1 (1.9)

Participants could select more than one reason from a list of possible reasons, or specify another reason

Participants reported a mean of 2.1 (SD = 2.8) emergency department visits in the 12 months prior to admission to custody, and more than three quarters of respondents (79/118) had at least one emergency department visit in that period. Those who did not have a family doctor or other primary care provider in the past 12 months reported a mean of 2.7 emergency department visits (SD = 3.8) in the past 12 months compared to a mean of 1.9 (SD = 2.3) in those who did have a family doctor or other primary care provider.

Almost two-thirds of participants indicated having at least one of the chronic conditions listed in the survey, and Table 3 shows the self-reported prevalence of each of these conditions. Of those who reported at least one of these chronic conditions, 30.3 % did not have family doctor or other primary care provider.

**Table 3:** Self-reported prevalence of chronic conditions, N = 125

Chronic condition	n (%)
Any of listed chronic conditions	81 (64.8)
Arthritis	19 (15.2)
Asthma	23 (18.4)
Chronic pain	21 (16.8)
Depression	52 (41.6)
Bipolar disorder, mania, manic depression, or dysthymia	26 (20.8)
Schizophrenia	6 (4.8)
Hepatitis C	23 (18.4)
Cancer	3 (2.4)
Diabetes	10 (8.0)
Heart disease including a heart attack	5 (4.0)
Stroke	2 (1.6)
High blood pressure or hypertension	15 (12.0)
HIV	3 (2.4)
Emphysema or COPD	3 (2.4)
Pregnant in the past year	4 (18.2)^a^

^a^Denominator used was 22, representing 21 participants who specified female gender and one participant who did not specify his or her gender but indicated having been pregnant in the past year

## Discussion

Persons in custody at a provincial correctional facility in southwestern Ontario report inadequate access to primary care, high unmet health care needs, and high emergency department use. Compared with the general Canadian population, study participants report worse access to primary care: 35 % in this study vs 15 % in the general population [36] did not have a primary care provider in the past 12 months. Study participants also have worse self-rated health [52], and more unmet health care needs (54 vs 8.8 %) [53, 54] and a higher mean number of emergency department visits (2.1 vs 0.3) [54, 55] in the past 12 months.

There is little coordination or continuity of care between health care in the correctional system and in the community in most jurisdictions in Canada [56]. Incarceration and detention may provide a unique opportunity to connect inmates with primary care and thus improve health care and health. In the United States, several regional programs that facilitate the transition from health care in custody to health care in the community have been described and studied [57–60]. A randomized controlled trial conducted in California from 2007 to 2009 demonstrated that older individuals and those with chronic conditions leaving prison will engage in primary care if provided early access. Moreover, the addition of a primary care-based care management program tailored for returning prisoners reduced emergency department use over expedited primary care [11]. In Canadian jurisdictions with large groups of persons being released from custody, linkage to a transitions clinic or other tailored primary care may be an effective intervention to improve health and to reduce costs associated with unnecessary emergency department use. To optimize uptake and effectiveness, any such interventions would need to address the barriers to primary care and the reasons why participants do not get their health care needs met, as identified in this study.

There are several potential limitations to this research. The response rate was low, which could affect the internal validity of the data. Comparing this study to others that were recently conducted in provincial facilities in Ontario [61, 62], one factor that may have affected the response rate is that the survey was provided in a written format instead of being interviewer-administered. An interviewer-administered format was not permitted by the Ministry because of the resources required to accommodate in-person interviews. This may have resulted in oversampling of persons with relatively high literacy, which may be associated with better access to health care and with lower morbidity [63]. We used a single question to assess access to primary care, which may not adequately represent participants’ primary care experience. We selected this measure because of widespread use in the general population [51] and feasibility in this short survey. Further, the study was conducted at a single provincial correctional facility, and the results may not be generalizable to persons in other provincial institutions in Ontario or in other provinces and territories; it is possible that access to primary care, health care needs, emergency department use, and the prevalence of chronic conditions varies by region or institution in Canada. The median age of our study population (33) is similar to the median age of those in sentenced custody (33) and on remand (31) in Ontario [54]. Moreover, given the consistency of our findings with international data, and given that (to our knowledge) no targeted programs exist to provide primary care in this population in Canada, we expect that the findings are likely true across persons in various correctional facilities across jurisdictions in Canada. Finally, these data are self-reported, which may introduce bias to estimates; however, any bias is unlikely to be large enough to nullify the large differences in findings for this population compared with the general population. In the future, we plan to examine health care utilization using health care administrative data to corroborate the findings of this study.

Improving access to health care and improving health in this population is an important public health and clinical priority [64], and may lead to benefits for the general population. The general population absorbs the increased health care costs of this population, and may be directly affected by the transmission of diseases such as hepatitis C. Further, imprisonment has been associated with increased levels of chronic diseases [65] and worse mental health [66] in the family members of those who are incarcerated [66]. At the population level, higher rates of incarceration have been associated with adverse health outcomes such as sexually transmitted infections and teen pregnancies [67]. The factors that lead to incarceration and subsequent recidivism, or repeat offence, are tightly linked to factors that affect health and wellbeing, and addressing underlying long-term health problems has been shown to reduce recidivism [68–70] and play a significant role in successfully reentering the community alongside other factors [71]. Finally, the right to health and health care is enshrined in international human rights documents [72, 73].

## Conclusions

Persons in a provincial correctional facility in Ontario, Canada have inadequate access to primary care, high unmet health care needs, and high emergency department use in the 12 months prior to admission. Time in custody may present an opportunity for connecting this population with primary care.

## Appendix

Appendix: includes a copy of the survey used in this study.
