# Erratum to: Access to primary care in adults in a provincial correctional facility in Ontario

**DOI:** 10.1186/s13104-016-2267-0

**Published:** 2016-10-11

**Authors:** Samantha Green, Jessica Foran, Fiona G. Kouyoumdjian

**Affiliations:** 1 Department of Family and Community Medicine, St. Michael’s Hospital, 30 Bond Street, Toronto, ON M5B 1W8 Canada; 2 Department of Political Science, McMaster University, Hamilton, ON Canada; 3 Centre for Research on Inner City Health, St. Michael’s Hospital, Toronto, ON Canada

## Erratum to: BMC Res Notes (2016) 9:131 DOI 10.1186/s13104-016-1935-4

Unfortunately, the original version of this article [1] was missing funding information within the Acknowledgements section.

The research for this article was funded by the Hamilton Community Foundation.
